# Ononin, a Natural Isoflavone Glycoside, Alleviates Postoperative Cognitive Dysfunction in Aged Mice by Regulating Neuroinflammation and Oxidative Stress

**DOI:** 10.1002/brb3.70952

**Published:** 2025-09-29

**Authors:** Ming Li, Qingmei Peng, Min Zhu, Qilin Liu, Simin Yang, Cansheng Gong, Jingyan Lin, Qingbo Yu

**Affiliations:** ^1^ Department of Anesthesiology the Affiliated Hospital of North Sichuan Medical College Nanchong China; ^2^ Department of Anesthesiology Suining Central Hospital Suining China; ^3^ Department of Anesthesiology Shengli Clinical Medical College of Fujian Medical University, Fujian Provincial Hospital Fuzhou China

**Keywords:** aging, neuroinflammation, ononin, oxidative stress, postoperative cognitive dysfunction

## Abstract

**Objective:**

Postoperative cognitive dysfunction (POCD) is a serious neurological complication that currently lacks effective clinical prevention and treatment. Ononin, a natural isoflavone glycoside, has been confirmed to exhibit potent neuroprotective effects. This study aimed to investigate whether ononin exerts a neuroprotective role against POCD.

**Methods:**

A POCD model was established in 18 months old mice with unilateral nephrectomy. Ononin (30 mg/kg) was administered intraperitoneally 15 min before surgery. On postoperative Day 3, the Morris water maze and open field tests were used to assess cognitive function. The pathological changes and apoptosis of hippocampal neurons were investigated by hematoxylin and eosin (HE) and terminal deoxynucleotidyl transferase dUTP nick end labeling (TUNEL) staining. In addition, western blotting and immunofluorescence staining were employed to examine the hippocampal levels of Iba1 and microglial activation. On Days 1 and 3 postsurgery, an enzyme‐linked immunosorbent assay (ELISA) was applied to gauge the expression of hippocampal IL‐1β, IL‐6, and TNF‐α. Meanwhile, the levels of hippocampal malondialdehyde (MDA), glutathione (GSH), and superoxide dismutase (SOD) were detected using the corresponding assay kits.

**Results:**

We found that anesthesia/surgery induced overt spatial memory deficits and neuronal damage in aged mice. Conversely, ononin pretreatment significantly rescued the pathological process and cognitive impairment. Mechanically, anesthesia/surgery triggered acute increases in hippocampal IL‐1β, IL‐6, TNF‐α, Iba1, and MDA, paralleled by a decline in SOD and GSH levels that was partially reversed by ononin.

**Conclusions:**

Our findings provide evidence that ononin may ameliorate anesthesia/surgery‐induced cognitive deficits through its anti‐inflammatory and antioxidant effects, which could be a novel preventive strategy for POCD.

## Graphical abstract

Ononin alleviates postoperative cognitive dysfunction in aged mice by regulating neuroinflammation and oxidative stress.

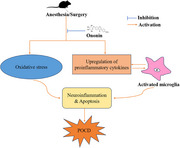

## 1 Introduction

Postoperative cognitive dysfunction (POCD) is a serious neurological complication following major surgery, especially in aged patients (Deiner and Silverstein 2009). The reported incidence of POCD ranges between 15%–60% (Evered and Silbert 2018; Urits et al. 2019). It is characterized by a decrease in learning, memory, concentration, or executive function following operation (Eckenhoff et al. 2020). Patients with POCD often exhibit reduced quality of life, prolonged hospital stays, and increased cost of hospitalization (Z. Li et al. 2022; Steinmetz et al. 2009). In addition, POCD may result in an increase in surgical morbidity and mortality (Terrando et al. 2011). Notably, cognitive impairment after anesthesia/surgical stress may not be transient, which could last for several years or even longer, potentially increasing the prevalence of Alzheimer's disease (AD) (Lin et al. 2020). As elderly people are expected to be the largest group undergoing surgery, their risk of developing POCD will increase significantly. Therefore, there is an urgent need to find ways to prevent the progression of POCD. Unfortunately, few effective methods have been excavated for the prevention and treatment of POCD to date.

Neuroinflammation (Z. Li et al. 2022), synaptic plasticity dysfunction (S. M. Chen et al. 2020), amyloid‐β (Aβ) deposition (Zhang et al. 2020), mitochondrial damage (Netto et al. 2018), and neuronal apoptosis (M. Li et al. 2019) have been proved to be implicated in the mechanisms of POCD. Nevertheless, an increasing number of researches have shown that among these mechanisms, neuroinflammatory reaction was the central link and played a pivotal role in the onset and development of POCD (Sun et al. 2022). Aseptic peripheral surgery or trauma could trigger immunological reactions, resulting in systemic release of pro‐inflammatory cytokines (Guo et al. 2021). Preclinical studies revealed that tumor necrosis factor α (TNF‐α), interleukin‐6 (IL‐6), and interleukin‐1β (IL‐1β), three major pro‐inflammatory factors, could cross the impaired blood–brain barrier (BBB) and enter the central nervous system, ultimately causing neuroinflammation and oxidative distress (K. Li et al. 2021; P. Wang et al. 2016). Further, microglia were activated, leading to the deterioration of neuroinflammation and oxidative damage (Q. Liu et al. 2021). Existing evidence suggests that the interaction between oxygen free radicals and pro‐inflammatory factors may exacerbate POCD (Godbout et al. 2005; Xie et al. 2021). Interventions that attenuated neuroinflammation and reactive oxygen species (ROS) could protect against cognitive impairment following surgery in aged mice (J. Liu, Gao, et al. 2023; Q. Liu et al. 2021; Safavynia and Goldstein 2018). Based on the above studies, treatments devoted to attenuating neuroinflammation and oxidative distress hold great potential in the prevention and treatment of POCD.

**FIGURE 1:**
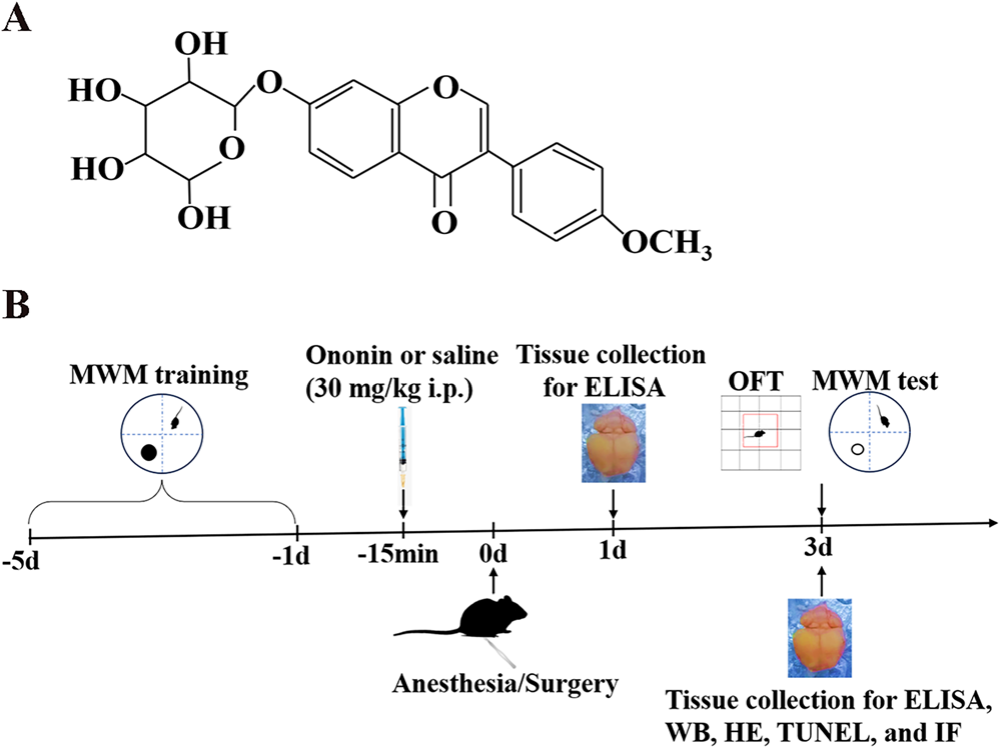
(A) The chemical structure of ononin. (B) Diagram of the experimental design. Seventy‐two aged mice were randomized into the surgery, surgery + ononin, control, and ononin groups. Prior to unilateral nephrectomy, all mice were subjected to water maze training for 5 consecutive days. Then, ononin or physiological saline (30 mg/kg) was administered to each mouse by intraperitoneal injection (i.p.) 15 min before surgery. On Day 3 postoperatively, the OFT and MWM tests were performed to assess exploratory locomotor activities and spatial memory, respectively. ELISA, enzyme‐linked immunosorbent assay, HE, hematoxylin and eosin, IF, immunofluorescence, MWM, Morris water maze, OFT, open field test, TUNEL, terminal deoxynucleotidyl transferase dUTP nick end labeling, WB, western blot.

Ononin (Figure 1A) is a natural and safe isoflavone glycoside, widely found in all sorts of traditional Chinese medicines, such as *Glycyrrhiza uralensis Fisch. ex DC*. (Fabaceae), *Pueraria montana (Lour.) Merr*. (Fabaceae), *Astragalus trimensis L*. (Fabaceae), and *Hedysarum spp. L*. (Fabaceae). Previous studies have demonstrated that ononin possesses pleiotropic biological activities, with the most focused on its anti‐inflammatory and antioxidant effects (X. Chen et al. 2021; Meng et al. 2021; Xu et al. 2022). X. Chen et al. (2021) found that ononin treatment exhibited potent neuroprotective effects in AD through inhibiting neuroinflammation and oxidative distress in rodents. In addition, Fu et al. (2014) uncovered that a structural analogue of ononin, calycosin‐7‐O‐β‐D‐glucoside could also afford neuroprotective effects in the middle cerebral artery occlusion (MCAO) ischemia‐reperfusion model in rats via regulating the NO/cav‐1/MMPs pathway. Of note, recent evidence suggests that ononin could ameliorate depression‐like behaviors in rats via regulating BDNF‐TrkB‐CREB signaling (Gong et al. 2024). Nevertheless, the therapeutic effect of ononin in POCD remains largely unclear. Consequently, the present study was conducted to determine whether ononin treatment could alleviate anesthesia/surgery‐induced cognitive deficits in elderly mice via suppression of neuroinflammation and oxidative stress.

## 2 Materials and Methods

### 2.1 Animals

Eighteen‐month‐old wild‐type C57BL/6 male mice, weighing between 30 and 36 g, were purchased from Sippe‐Bk Lab Animal Company (Shanghai, China). Five individuals per cage were housed in a specific‐pathogen‐free (SPF) room (23 ± 1°C) and allowed to freely obtain food and water under 12–12 h light–dark cycles. The humidity of the SPF room was kept at 60 ± 5%. Before the experiment, mice were permitted for adaptation of the laboratory circumstance for 7 days. Our study was approved by the Ethics Committee of North Sichuan Medical College Institutional (dated: 08/06/2022). All procedures were executed in line with the “Guide for the Care and Use of Laboratory Animals” published by the NIH.

### 2.2 Experimental Design

Animals were randomly (computer‐based randomization) allocated into the following groups (*n* = 18 per group): control, surgery, ononin, and surgery + ononin groups (surgery + ononin). Ononin (Sigma–Aldrich, USA) was dissolved in saline containing 1% DMSO (Solarbio, China). The ononin and the surgery + ononin groups received ononin by intraperitoneal injection (30 mg/kg) (Fu et al. 2014) 15 min before onset of surgery. At the corresponding time point, the other two groups were administrated to an equal volume of saline containing 1% DMSO. The schematic diagram of the experimental design is described in Figure 1B.

### 2.3 Animal Model of POCD

Unilateral nephrectomy is a well‐established method for the development of a mouse POCD model (Du et al. 2019; C. Liu, Wu, et al. 2023). Thus, as our previously described approach (C. Chen, Gao, et al. 2019), unilateral nephrectomy was carried out to generate a POCD model in aged mice. Briefly, mice were inducted with 4% sevoflurane through a conical mask (2% in oxygen), followed by 2%–2.5% for maintenance. Body temperature during the procedure was kept between 36.5°C and 37.5°C utilizing a heating pad. Then, we carefully shaved off the hair in the surgical area and thoroughly cleaned it with 75% ethanol. A transverse incision of approximately 1.5 cm was made and the left kidney was exteriorized and carefully resected. For postoperative analgesia, a subcutaneous injection of 200 µL ropivacaine (0.2%) was used. The researchers, who were aware of the detailed grouping of the mice, took responsibility for collecting experimental samples, but the evaluators of the experimental results were blinded to the allocation of the mice groups. The entire surgical procedure took approximately 40 min. Mice that did not undergo surgery received only a 1:1 mixture of air and 100% oxygen treatment for the same period of time.

### 2.4 Open Field Test

Open field test (OFT) is used to estimate the autonomous activity and spatial exploration in rodents. OFT was conducted on Day 3 postoperatively based on our previous study (M. Li et al. 2019) with slight modifications. Mouse was placed gently into the center (50% of the total area) (Xue et al. 2022) of the OFT device (50 × 30 × 25 cm^3^), and allowed free exploration for 5 min. A video camera was employed for automatically recording the exploratory locomotor activities. The total travel distance, mean velocity, rearing activity, and duration in the center were analyzed by means of a maze scan software (Top‐view v.3.0, Cleversys, USA). After each test, the OFT apparatus was cleaned with 75% alcohol.

### 2.5 Morris Water Maze Test

Morris water maze (MWM) is a classical and reliable means for the evaluation of spatial learning and memory in rodents (Vorhees and Williams 2006). As described previously (Da Mesquita et al. 2018), the MWM test was executed with minor modifications. In brief, prior to the start of the test, mice were transported into the test room for habituation at least 30 min. A circular tank pool with a 50 cm depth and 120 cm diameter was filled to a depth of 30 cm with opaque water made by adding white and nontoxic dye. We maintained the water temperature at 23 ± 1°C and divided the tank pool into an average of four quadrants. A dim light was put into the test room, and four different shapes of visual cues were placed above each quadrant of the pool to assist the mice to navigate and locate the submerged platform. A platform with a diameter of 10 cm was hidden 1 cm below the water surface and placed in the middle of one quadrant. The platform remained unchanged throughout the training phase and was removed during the probe testing. A video camera was installed above the pool and connected to the maze scan software (Cleversys, USA) for tracing and analyzing the animal's movement. In the training session (acquisition), four consecutive trials per day were conducted on each mouse for 5 days before the operation (Figure 1B). Each mouse facing the tank wall was put into the water in the desired starting position. The time limit for mice to find and climb onto the platform was 60 s. If the mice could not climb onto the platform in the allotted time, we gently placed them on the platform and let them stay for 20 s. During the acquisition, the swimming speed and the latency to the platform were calculated for each day. On postoperative Day 3, the platform was removed. In the opposite quadrant, mice were released and tested for 60 s in the probe testing. For this session, the latency to the previous platform, the percentage of time in the platform quadrant, and the entries across the previous platform were recorded and analyzed.

### 2.6 Brain Tissue Preparation

Mouse hippocampal tissues were harvested on Days 1 and 3 after surgery. On Day 1 postsurgery, six mice from each group were anesthetized with a lethal dose of sodium pentobarbital (100 mg/kg, intraperitoneal injection), and then transcardially perfused with ice‐cold 0.9% saline. The brain tissue was swiftly extracted with ophthalmic scissors and forceps. Subsequently, the hippocampus was carefully isolated and transferred into a 1.5 mL freezing EP tube, followed by storage at −80°C in a refrigerator for subsequent assays, including enzyme‐linked immunosorbent assay (ELISA) and anti‐oxidative enzyme activity assay, respectively. On Day 3 after surgery, the remaining mice were euthanized after the end of behavioral test using the same method. The hippocampus was collected for examination of inflammatory factors and oxidative stress response levels, as well as for hematoxylin and eosin (HE) staining, terminal deoxynucleotidyl transferase dUTP nick end labeling (TUNEL) assay, immunofluorescence (IF), and western blot (WB) analysis.

### 2.7 Oxidative Distress Determination

According to the manufacturer's instructions from Nanjing Jiancheng Bioengineering Institute, the antioxidant enzymes superoxide dismutase (SOD) assay kit, nonenzymatic antioxidant glutathione (GSH) assay kit, and the oxidative product malondialdehyde (MDA) assay kit (Nanjing, China) were utilized to measure hippocampal SOD activity, GSH, and MDA levels. A microplate reader (VirusScan LUX, USA) was employed to detect the corresponding OD values (MDA at 532 nm, GSH at 405 nm, SOD at 450 nm). Based on the formula in the kit manual, MDA (nmol/mg protein) and GSH (µmol/g) levels, as well as SOD activity (U/mg protein) were calculated.

### 2.8 Determination of Inflammatory Markers

The hippocampus was obtained on Days 1 and 3 after unilateral nephrectomy. We employed ELISA kits (Fine Test, #EM0183, #EM0109, and #EM0121, China) to determine the levels of TNF‐α, IL‐6, and IL‐1β in the light of the manufacturer's protocols.

### 2.9 HE Staining

After the end of behavioral test, the mice were anesthetized with sevoflurane. When the depth of anesthesia was sufficient to meet the surgical requirements, the thoracic cavity was opened and 20 mL of cold 0.9% saline was perfused through the left ventricle for 3 min, followed by 20 mL of 4% paraformaldehyde (PFA) for 10 min. The brains were immediately removed and immobilized in 4% PFA at 4°C for 48 h, followed by routine dehydration and paraffin embedding. Then, each sample was sectioned at a thickness of 3 µm. After dewaxing and rehydration, the paraffin slice was subjected to standard HE staining (Solarbio, China) according to the manufacturer's instructions. Finally, the hippocampal CA1 and DG regions in each sample were observed using an optical microscope (Olympus BX53, Japan).

### 2.10 TUNEL Assay

The apoptosis in the hippocampus was determined using a TUNEL apoptosis detection kit (Cellorlab, Shanghai, China) according to the manufacturer's instructions. In brief, as with the HE staining, the brain slices of 3 µm thickness were prepared. After drying, dewaxing, and hydration, the slices were permeabilized with proteinase K for 10 min in a black wet box at 37°C and then washed with PBS three times. Afterwards, each sample was subjected to a treatment with 50 µL of TUNEL reaction solution, followed by incubation in the wet box at 37°C for 60 min. After being washed with PBS twice, the samples were treated with DAPI solution (Solarbio, #2871890‐3, China) for nuclear staining. TUNEL‐positive nuclei that presented both the green and blue signals under a fluorescence microscope (Olympus FV1200, Tokyo, Japan) from five different microscopic fields in each section of hippocampal CA1 region (400× magnification) were identified and counted, and the percentage of TUNEL‐positive nuclei was calculated.

### 2.11 IF Analysis

Ionized calcium binding adapter molecule 1 (Iba1), an important marker of microglia, was immunofluorescence stained in accordance with the antibody manufacturer to investigate microglial morphology. Briefly, mouse was deeply anesthetized, and then subjected to transcardiac perfusion with 25 mL of 0.9% saline, followed by 20 mL of 4% paraformaldehyde (PFA). After that, the brain was removed, fixed by 4% PFA for 24 h, and embedded in the thick paraffin. Then, the brain was dissected at a thickness of 3 µm. The slices were subjected to dewaxing, rehydration, and antigen repair in sequence. Thereafter, donkey serum (10%) (Solarbio, China) was utilized to block the nonspecific binding sites at 37°C for 1 h. The slices were then incubated with the primary antibody (rabbit anti‐Iba1, #ab178846, Abcam, 1:100, UK) overnight at 4°C. Subsequently, the slides were briefly washed with phosphate balanced solution three times for 5 min, followed by incubation with secondary antibody (Abcam, #ab150077, 1:200, UK) for 1 h at room temperature. Then, DAPI was applied for counterstaining nuclei for 5 min. Through an Olympus confocal BX‐60 imaging system (Tokyo, Japan), the images of hippocampal CA1 region in aged mice were obtained at 400× magnification. The average number and fluorescence intensity of Iba1‐positive cells were manually analyzed and calculated from five different microscopic fields in each section of the hippocampal CA1 region using ImageJ software (National Institutes of Health, USA).

### 2.12 WB Analysis

WB was conducted based on our previous research (M. Li et al. 2019). In brief, equal amounts (100 µg of protein) of the sample were separated using 15% SDS‐PAGE gels, and then transferred to activated PVDF membranes (0.45 µm) (Millipore, USA). Afterwards, at room temperature, the membranes were blocked for 1 h with 5% non‐fat milk solution, and incubated overnight at 4°C by the corresponding primary antibodies against Iba1 (Abcam, #ab178846, 1:1000, UK) and GAPDH (Proteintech, #10494‐1‐AP, 1:3000, China). After three washes with TBST, the membranes were incubated for another 1 h at room temperature with HRP‐conjugated secondary antibodies (Zsbio, #ZB‐2306, 1:3000, China). The targeted protein band was visualized through employing an Enhanced Chemiluminescence Kit (Millipore, #WBKLS0100, USA). Bands were quantified using Image J 8.0.

### 2.13 Data Analysis and Statistics

All data were presented as mean ± SEM. Prism 9.0 software (GraphPad software, USA) was applied to perform statistical analyses. Normality of the experimental data was evaluated by the Shapiro–Wilk test. The data of MWM were analyzed by two‐way analysis of variance (ANOVA) followed by the Tukey post hoc test for repeated measures. For other data, such as OFT, quantification of immunoreactivity, and normalized band intensities in western blot, we used one‐way ANOVA followed by the Tukey post hoc test for the assessment of statistical significance. Differences with *p* < 0.05 were considered statistically significant.

## 3 Results

### 3.1 Unilateral Nephrectomy Imposed a Negligible Effect on the Spontaneous Locomotor Activities

**FIGURE 2:**
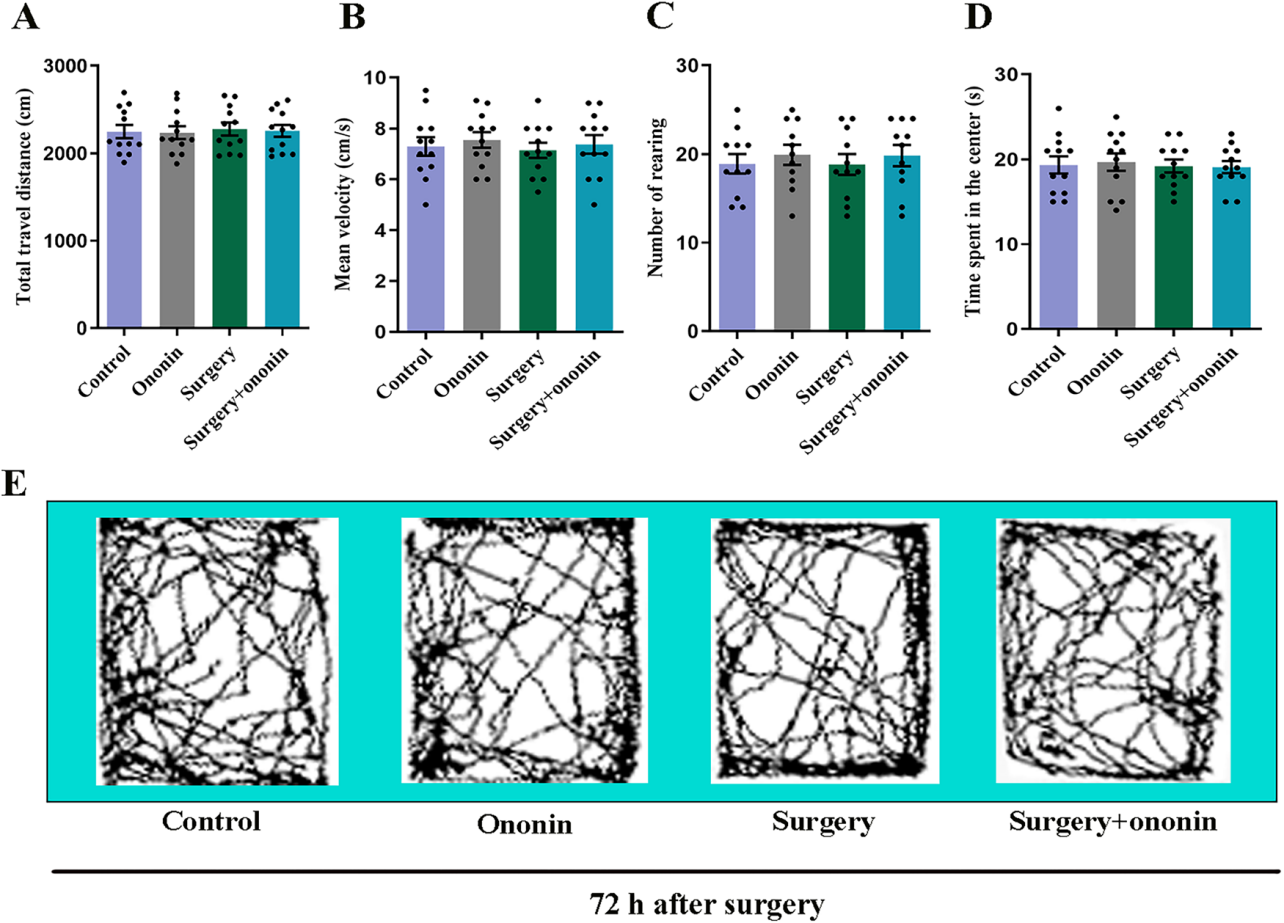
The spontaneous locomotor activities in elderly mice were not affected by unilateral nephrectomy. (A) Total travel distance. (B) Mean velocity. (C) Rearing activity. (D) Duration in the center. (E) Representative motion trajectories in the open field device for the four groups. *n* = 12 per group.

We employed the OFT for the evaluation of spontaneous locomotor activities of aged mice on Day 3 postoperatively. Our results indicated that there was little difference between groups (*p >* 0.05, *n* = 12/group) with regard to total travel distance (Figure 2A), mean velocity (Figure 2B), rearing activity (Figure 2C), and duration in the center (Figure 2D,E). These data suggest that unilateral nephrectomy had negligible influences on the spontaneous locomotor activities, and the cognitive dysfunction in surgery‐treated mice was unlikely to be due to impaired spontaneous locomotor ability.

### 3.2 Ononin Prophylaxis Preserved Learning and Memory Following Unilateral Nephrectomy

**FIGURE 3:**
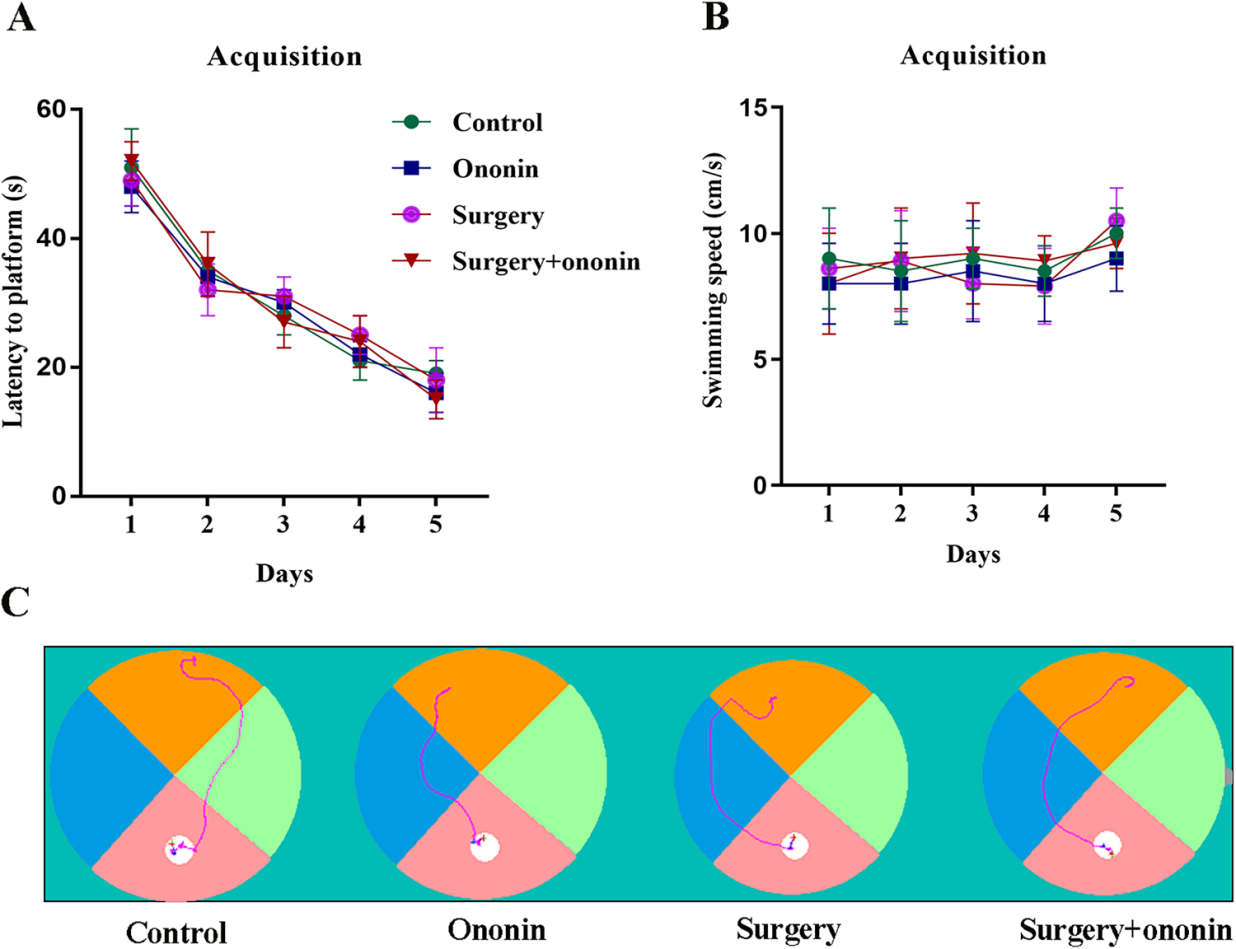
Performance of the aged mice in the MWM training phase. (A) Average escape latency. (B) Swimming speed. (C) Representative motion traces of aged mice after 5 consecutive days of training in the MWM. *n* = 12 per group.

**FIGURE 4:**
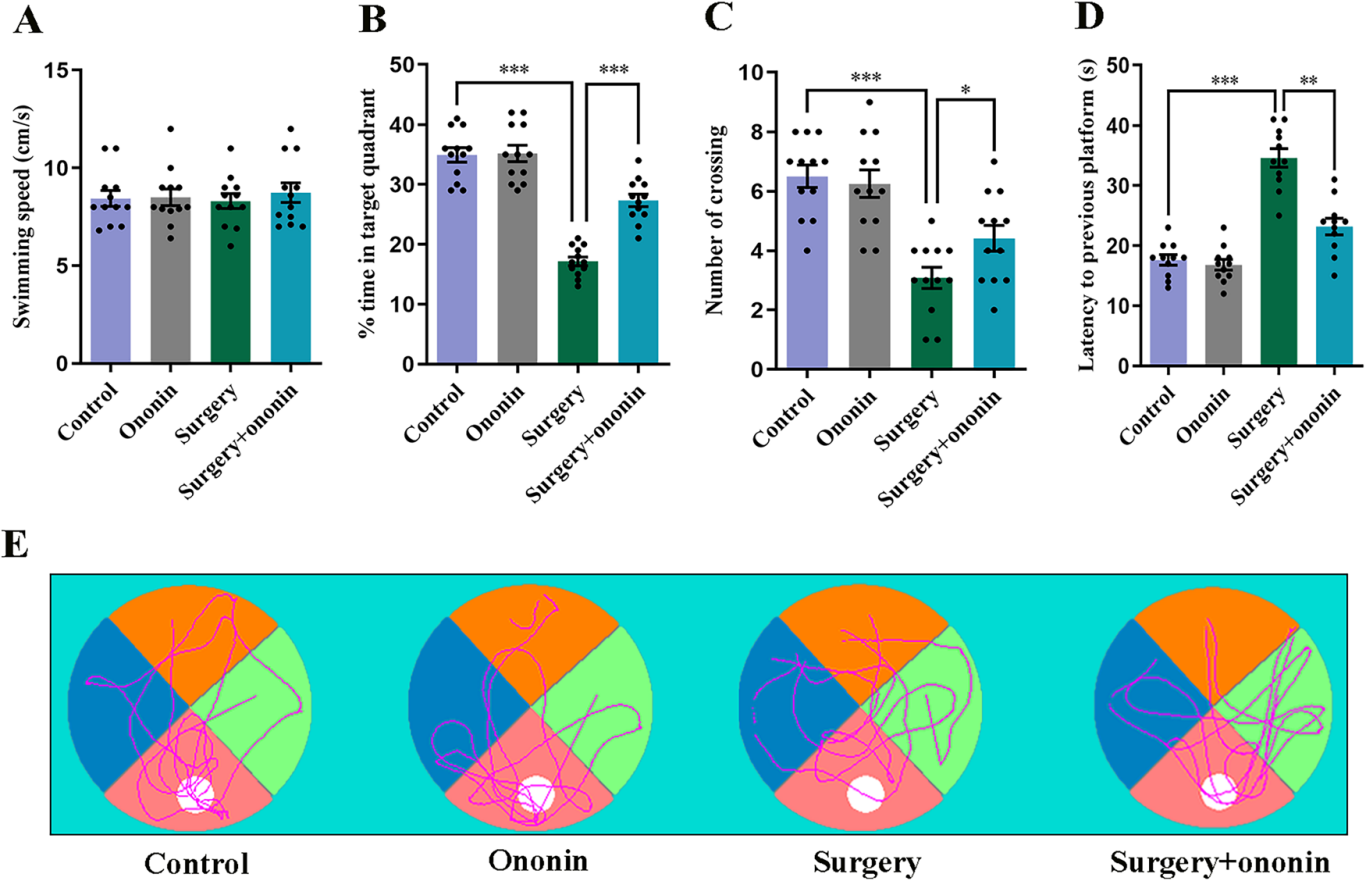
Ononin improved learning and memory dysfunction following surgery in aged mice. The ability of motor and memory was evaluated in the probe trial by analyzing the swimming speed (A), time spent in the target quadrant (B), number of crossing platforms (C), and latency to the previous platform (D). (E) Representative swimming paths. *n* = 12 per group^. *^ *p* < 0.05, ^**^p < 0.01, ^***^p < 0.001.

In the acquisition of the MWM test, the spatial learning and memory abilities of elderly mice improved after training over time, and no differences were detected in the average swimming speed and escape latency between groups (*p* > 0.05, *n* = 12/group, Figure 3A–C). During the probe testing, there was also little difference among the four groups in terms of average swimming speed (*p* > 0.05, *n* = 12/group, Figure 4A). However, the number of crossing platforms and the percentage of time spent in the target quadrant were significantly reduced in surgery‐treated mice on Day 3 postoperation when compared to the controls (*p* < 0.001, *n* = 12/group, Figure 4B,C). On the contrary, the latency to the previous platform increased markedly in the surgery group when compared to the control group (*p* < 0.001, *n* = 12/group, Figure 4D,E), suggesting that unilateral nephrectomy may cause learning and memory deficits in elderly mice. Importantly, anesthesia/surgery‐induced cognitive decline was notably ameliorated by ononin pretreatment (*p* < 0.05, *n* = 12/group, Figure 4B–E).

### 3.3 Ononin Alleviated Hippocampal Neuronal Damage Induced by Anesthesia/Surgery

**FIGURE 5:**
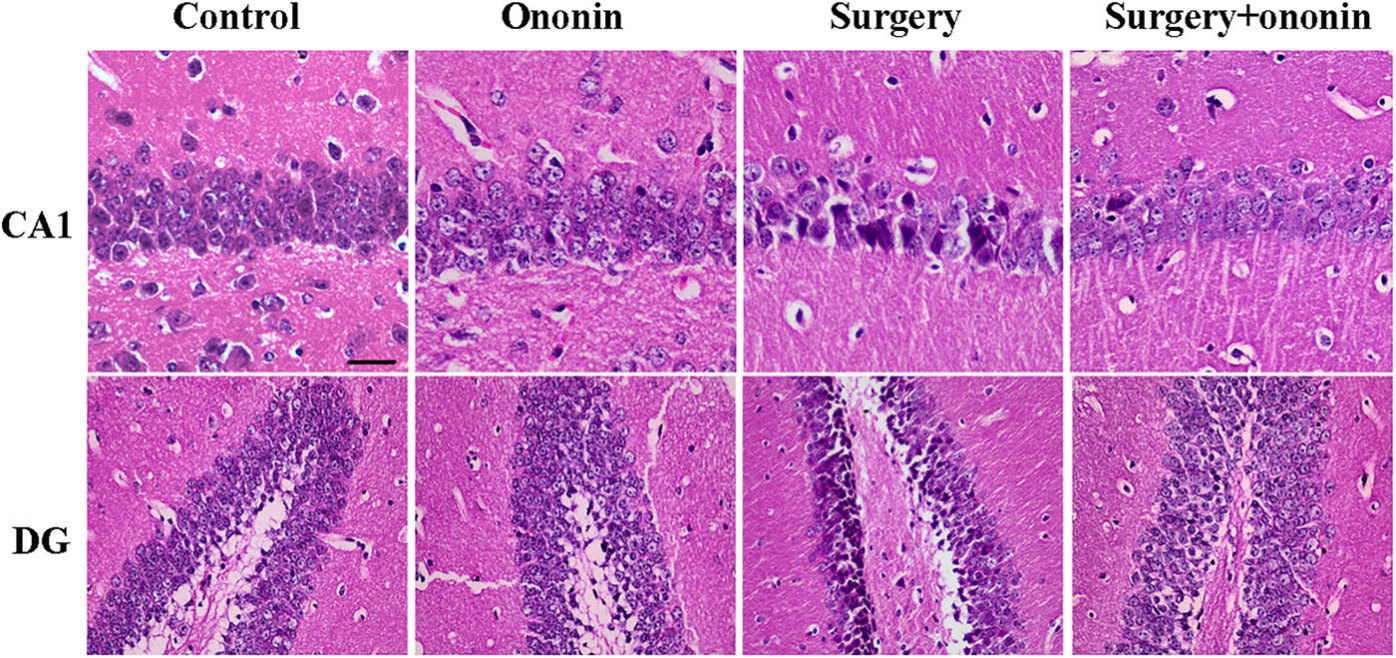
Treatment with ononin significantly reversed the pathological damage in hippocampal neurons. On Day 3 postsurgery, the pathological changes of the hippocampal CA1 and DG neurons by HE staining were observed under an optical microscope (400×). Scale bar, 50 µm. *n* = 6 per group.

**FIGURE 6:**
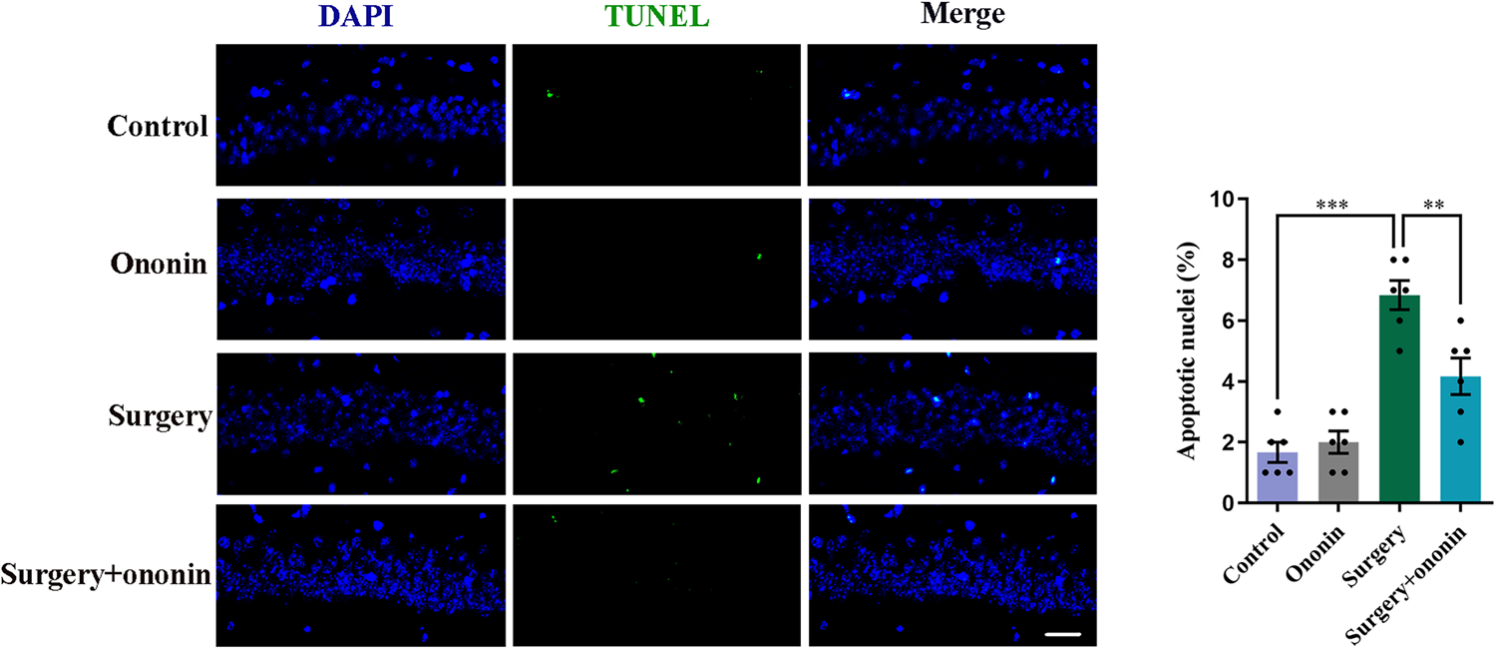
Ononin prophylaxis diminished hippocampal apoptosis induced by unilateral nephrectomy. Representative images of TUNEL staining in the hippocampal CA1 region and the quantification of TUNEL‐positive nuclei on Day 3 after surgery (400×). Scale bar, 20 µm. *n* = 6 per group.

The HE staining results showed that the neurons in the CA1 and DG regions of the hippocampus in the control and ononin groups exhibited normal morphology, characterized by clear boundaries, well‐organized arrangement, and uniform staining (*n* = 6/group, Figure 5). However, the hippocampal neurons of the surgery group mice displayed a significant damage, with an unclear cellular structure, disordered arrangement, deeply stained nuclei, and a marked decrease in cell density (*n* = 6/group, Figure 5). Surprisingly, preoperative treatment with ononin contributed to a more regular arrangement of hippocampal neurons, less deeply stained nuclei, and an obvious increase in cell density when compared with the surgery group (*n* = 6/group, Figure 5). Next, to detect apoptosis in the hippocampus, TUNEL staining of brain sections was conducted. In comparison to the control group, TUNEL‐positive nuclei in the hippocampal CA1 region were significantly increased in the surgery group, whereas ononin pretreatment dramatically reversed the effect (*p* < 0.01, *n* = 6/group, Figure 6). These data suggest that ononin prophylaxis affords a notable improvement in hippocampal cell damage after anesthesia/surgery.

### 3.4 Ononin Attenuated Anesthesia/Surgery‐Triggered Increases in Hippocampal Pro‐Inflammatory Cytokines

**FIGURE 7:**
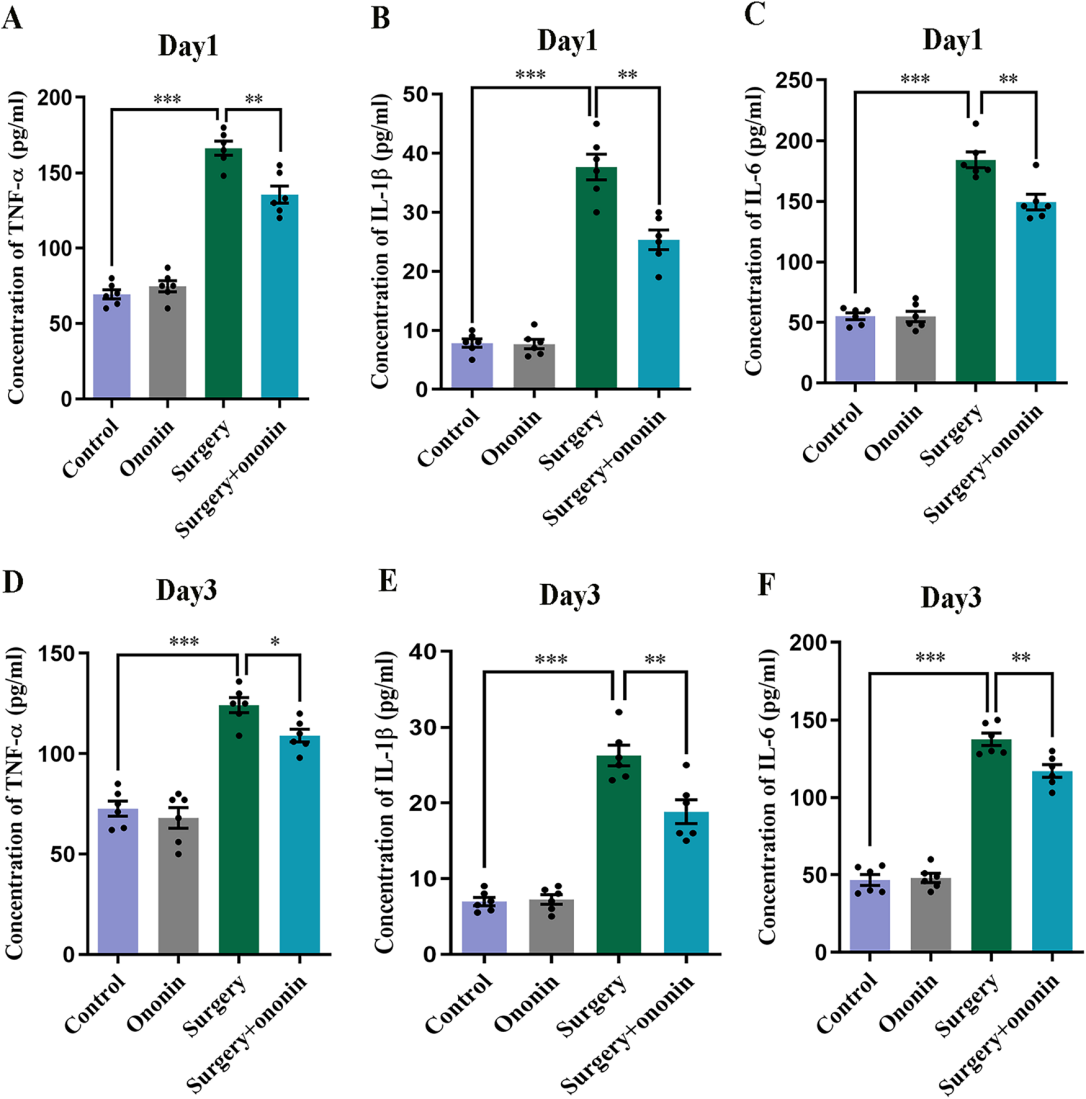
Ononin pretreatment attenuated unilateral nephrectomy‐triggered hippocampal neuroinflammation. The hippocampal levels of TNF‐α, IL‐1β, and IL‐6 on Days 1 (A–C) and 3 (D–F) postoperatively in elderly mice. *n* = 6 per group.

Neuroinflammation related to surgical trauma is a critical pathogenic element for the development of POCD (Y. Chen et al. 2023). Thus, to determine whether the therapeutic role of ononin on cognitive defect was linked to the reduced neuroinflammation, we measured the content of pro‐inflammatory cytokines (IL‐6, TNF‐α, and IL‐1β) in the hippocampus, a vital brain region associated with cognition (Pilarzyk et al. 2023). As shown in Figure 7A–F, on postoperative Days 1 and 3, these three cytokines in the hippocampal tissues of surgery‐treated mice were considerably elevated (*p* < 0.001, *n* = 6/group), while this increase was significantly reversed at both time points by application of ononin (*p* < 0.05, *n* = 6/group). These findings verified that ononin pretreatment could dampen anesthesia/surgery‐induced hippocampal inflammation.

### 3.5 Ononin Reversed Anesthesia/Surgery‐Induced Hippocampal Microglial Activation

**FIGURE 8:**
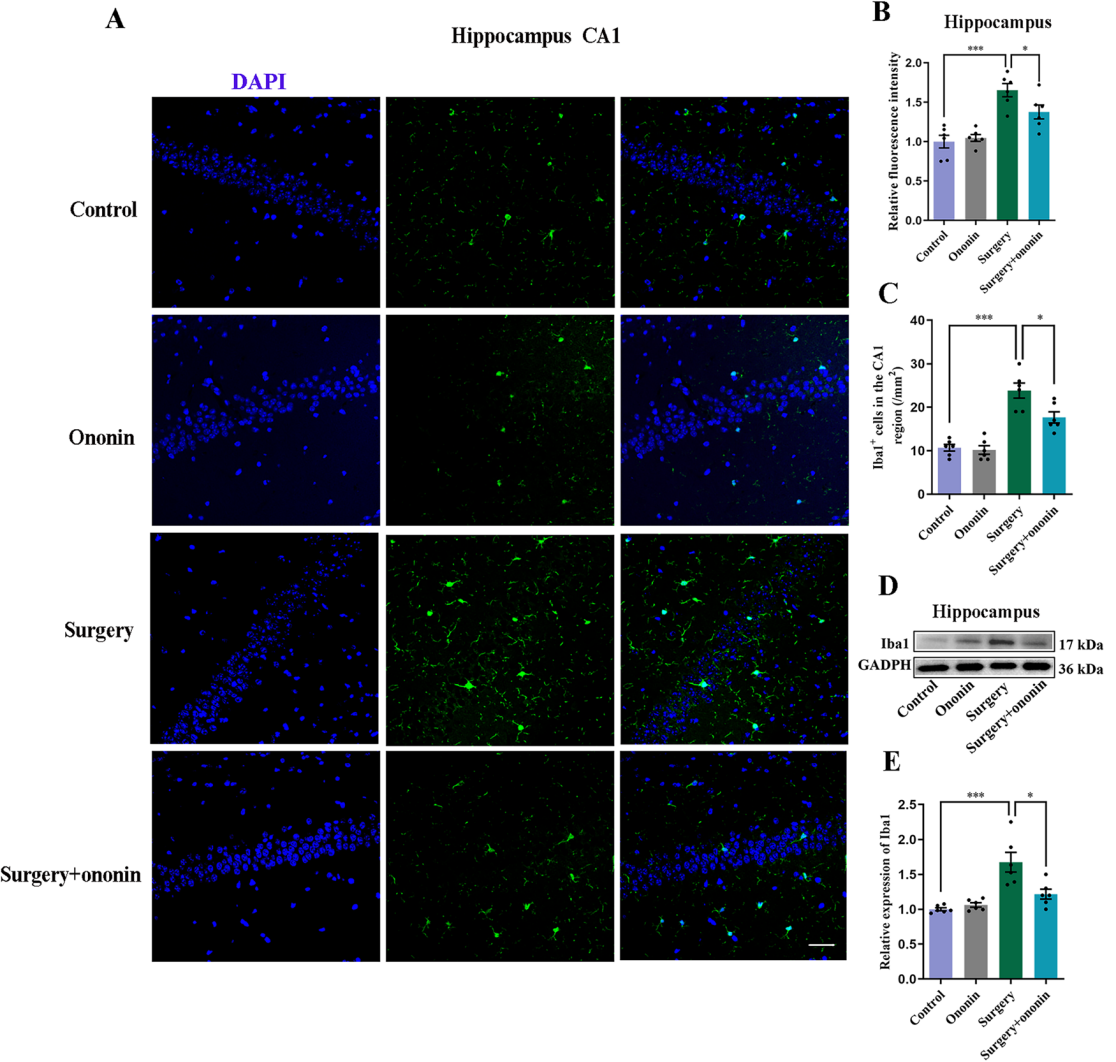
Ononin mitigated hippocampal microglial activation elicited by anesthesia/surgery. (A) Representative images of Iba1 at 72 h after unilateral nephrectomy in the hippocampal CA1 region of elder mice. (B) Quantification of Iba1 fluorescence intensity. (C) The number of Iba1‐positive cells in the CA1 area. (D) Representative blots of Iba1 on Day 3 postoperation. (E) Quantification of Iba1 protein. Scale bar, 20 µm. *n* = 6 per group.

Activated microglia exert a crucial role in surgery‐elicited neuroinflammation and neuronal damage (Gao et al. 2023). Therefore, to investigate hippocampal microglial activation in aged mice, we utilized immunofluorescence staining and western blotting to examine the levels of Iba1, a reliable cytoplasmic microglial marker with a strong signal. Our data showed that the number and average fluorescence intensity of Iba1‐positive cells in the hippocampal CA1 region were obviously increased in the surgery‐treated mice when compared to the controls (*p* < 0.001, *n* = 6/group, Figure 8A–C). Meanwhile, the Iba1‐positive cell bodies displayed marked hypertrophy after surgery (Figure 8A). We also found that the protein levels of Iba1 were dramatically enhanced in the surgery group when compared to the control group (*p* < 0.001, *n* = 6/group, Figure 8D,E). By contrast, ononin pretreatment significantly attenuated the anesthesia/surgery‐induced alteration of Iba1and microglia (*p* < 0.05, *n* = 6/group, Figure 8A–E).

### 3.6 Ononin Ameliorated Anesthesia/Surgery‐Elicited Hippocampal Oxidative Distress Response

**FIGURE 9:**
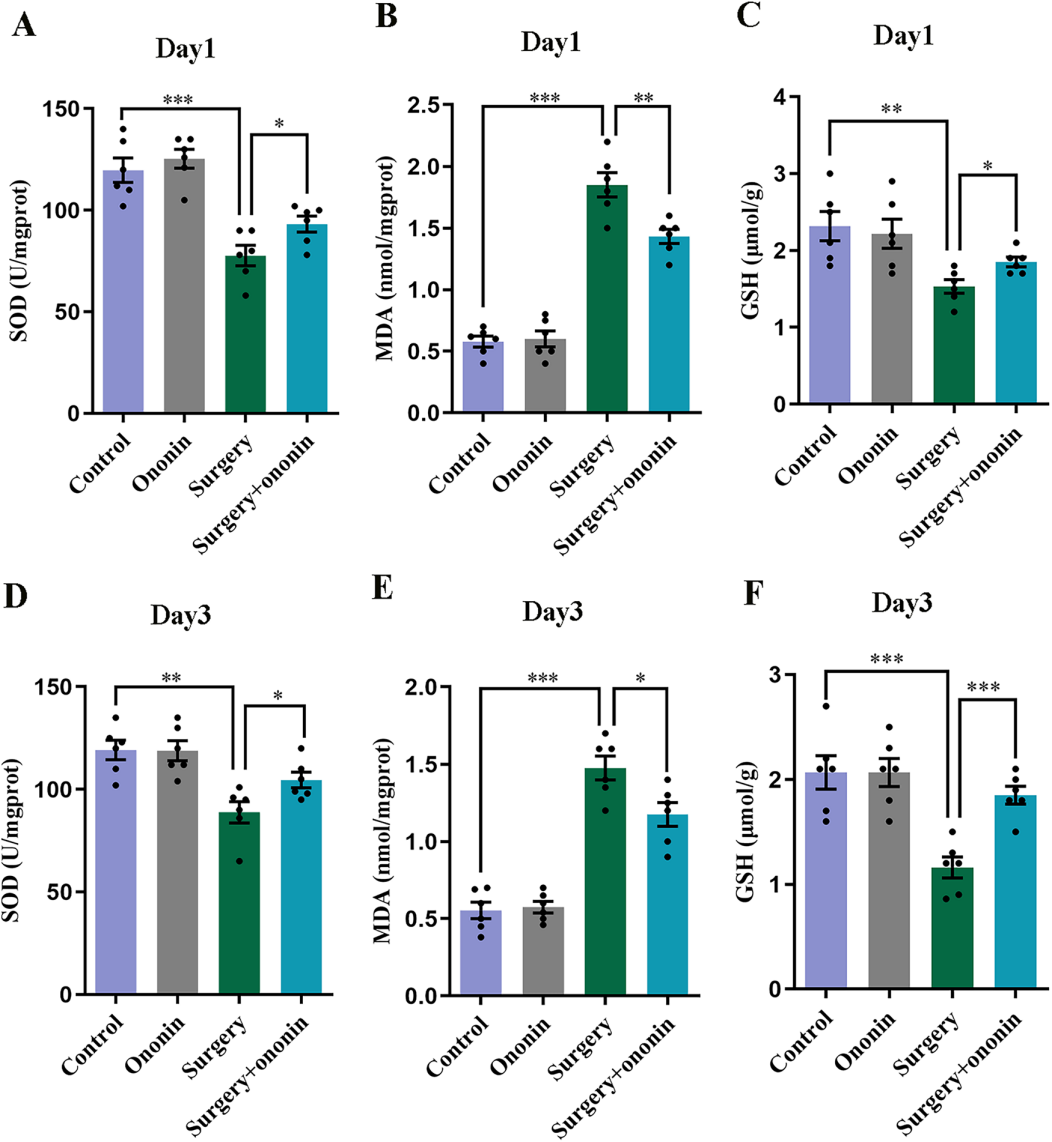
Ononin attenuated hippocampal oxidative distress caused by anesthesia/surgery. SOD activity, GSH and MDA levels in the hippocampus of elderly mice on postoperative Days 1 (A–C) and 3 (D–F). *n* = 6 per group.

The excessive mitochondrial oxidative stress response also contributed to increased susceptibility to POCD (Wang et al. 2023), we therefore determined whether ononin could exert neuroprotective effects by alleviating oxidative distress. As expected, the levels of SOD and GSH in the hippocampus were considerably reduced in the surgery‐treated mice on postoperative Days 1 and 3 relative to the controls (*p* < 0.01, *n* = 6/group, Figure 9). However, the decreased SOD and GSH levels after surgery were largely rescued by ononin administration (*p* < 0.05, *n* = 6/group, Figure 9). In contrast, MDA levels were greatly elevated on Days 1 and 3 postsurgery (*p* < 0.001, *n* = 6/group, Figure 9) in the hippocampus of surgery‐treated mice when compared with the controls. Likewise, ononin pretreatment partially reversed the postoperative increase in hippocampal MDA levels (*p* < 0.05, *n* = 6/group, Figure 9).

## 4 Discussion

In this study, we found that the hippocampal neuronal damage and cognitive deficits in elderly mice could be elicited by unilateral nephrectomy on postoperative Day 3. Importantly, the effects of anesthesia/surgical stress were notably reversed by ononin pretreatment. Mechanically, we demonstrated that ononin could remarkably attenuate unilateral nephrectomy‐induced hippocampal neuroinflammation and oxidative distress in elderly mice. As a result, our findings provide preclinical evidence that ononin may act as a promising therapeutic drug for anesthesia/surgery‐triggered cognitive impairment via mitigating hippocampal inflammation and oxidative insults.

Previously, several POCD models in rodents have been established to mimic the condition that patients may suffer from cognitive impairment following surgery, including exploratory laparotomy (J. Liu, Gao, et al. 2023), tibial fracture surgery (Zhou et al. 2023), and excision of important organs under anesthesia (Vizcaychipi et al. 2014). In the current study, unilateral nephrectomy in aged mice was conducted under sevoflurane anesthesia to set up a POCD model. As demonstrated in previous reports (J. Liu, Gao, et al. 2023; Vizcaychipi et al. 2014), we corroborated that this surgical approach could elicit early postoperative cognitive impairment in aged mice. Meanwhile, the aged mice that underwent unilateral nephrectomy did not alter mean velocity and total travel distance relative to the controls in the OFT, indicating that the cognitive deficits in surgery‐treated mice were not attributed to the impairment of spontaneous locomotor ability. Also, we found that our protocol had no impact on the kidney function and mortality in aged mice after surgery (data not shown), which is consistent with the previously published literature (C. Chen et al. 2015) and in line with clinical practice.

The MWM is a classical approach for the evaluation of hippocampal‐dependent learning and memory in rodents (Da Mesquita et al. 2018). In this study, after 5 consecutive days of MWM training, the aged mice in each group were able to find the platform quickly and possessed similar spatial memory in the MWM before surgery. On Day 3 postsurgery, mice exposed to anesthesia/surgery manifested memory deficits, as evidenced by increased latency to the previous platform and decreased percentage of time spent in the target quadrant, as well as a reduced number of crossing platforms compared to the controls. These results are in agreement with previous studies (Wang et al. 2023; Xie et al. 2021). Of note, preoperative intervention with ononin significantly reversed the water‐maze impairment in aged mice, denoting that ononin could ameliorate cognitive function following anesthesia/surgery.

A growing body of research has demonstrated the crucial role of neuroinflammation in the pathophysiology of POCD (Jiang et al. 2023). Q. Liu et al. (2021) found that suppression of hippocampal neuroinflammation by Sirtuin 3 protected elderly mice from anesthesia/surgery‐induced cognitive impairment. Likewise, a large number of preclinical studies unveiled that administration of anti‐neuroinflammatory drugs, such as dexmedetomidine (Xie et al. 2021), fluoxetine (Yao et al. 2023), and omega‐3 fatty acids (Guo et al. 2021), could relieve surgery‐triggered neuroinflammation and preserve learning and memory abilities in aged rodents. In addition, it is well‐documented that microglia can exert a key role in immune regulation and phagocytosis of dangerous cells or materials (Y. Liu et al. 2022). Surgery‐evoked elevations of hippocampal pro‐inflammatory cytokines can further hyperactivate microglia and exacerbate neuroinflammation (Teeling and Perry 2009). Activated microglia can elicit neurotoxicity, resulting in damage to synapses and neurons (L. Chen, Dong, et al. 2019; Zeng et al. 2022). Our results revealed that hippocampal levels of TNF‐α, IL‐1β, and IL‐6 were markedly elevated on Days 1 and 3 postsurgery, accompanied by overt activation of Iba1‐labelled microglia, which was consistent with previous researches (M. Li et al. 2019; Q. Liu et al. 2021). By contrast, ononin pretreatment attenuated anesthesia/surgery‐triggered increases in hippocampal pro‐inflammatory cytokines and reversed microglial activation to some degree. Thus, the improvement of anesthesia/surgery‐induced cognitive decline by ononin may be closely related to its anti‐neuroinflammatory properties.

Clinical evidence has uncovered that advanced age is one of the main risk factors of developing POCD (Miller et al. 2018; Travica et al. 2023). As individuals age, oxidative stress in the brain becomes increasingly evident, characterized by increased ROS levels and decreased antioxidant enzymes SOD activity (Santos and Sinha 2023). Oxidative imbalances can cause the accumulation of harmful proteins and dysfunctional mitochondria in elderly patients, which is another pivotal factor contributing to POCD (Kaushik et al. 2021; Wang et al. 2023). Crucially, oxidative distress and neuroinflammation coexist and interact, further exacerbating anesthesia/surgery‐induced cognitive impairment (L. Li et al. 2023; Xie et al. 2021). Accumulating studies have revealed that ononin possessed potent antioxidant effects (Ye et al. 2022). For example, a recent study demonstrated that ononin treatment could mitigate cognitive impairment in AD rats elicited by aluminum chloride through suppressing oxidative distress and neuroinflammation (X. Chen et al. 2021). In addition, Yan et al. (2019) found that ononin could effectively alleviate OGD/R‐evoked HT22 cell damage and apoptosis by suppressing oxidative distress. This knowledge inspired us to explore the latent benefits of ononin on oxidative insults and cognitive decline induced by anesthesia/surgery. In concordant with previous researches (P. Wang et al. 2016), we unveiled that unilateral nephrectomy could trigger hippocampal oxidative distress in surgery‐treated mice, as evidenced by apparently reduced SOD and GSH levels and elevated MDA content on Days 1 and 3 postsurgery. Not surprisingly, ononin prophylaxis appreciably reversed these changes, which was in accordance with the decreased neuroinflammation in the hippocampus. This coincides with the extensively believed antioxidant characteristics of ononin in several neurological diseases, such as AD (X. Chen et al. 2021). Accordingly, these findings suggest that the neuroprotective role of ononin in POCD may be implicated in its antioxidant effects as well.

In our study, the reason why we selected the dosage of 30 mg/kg ononin and injected it intraperitoneally into aged mice 15 min before surgery was based on the safety and efficacy of ononin application. A previous study (Fu et al. 2014) showed that a representative component of isoflavones in *Astragalus trimensis L*. (Fabaceae), calycosin‐7‐O‐β‐D‐glucoside, which is a structural analogue of ononin, administered intraperitoneally 15 min prior to cerebral ischemia could afford neuroprotective effects in a rat model of MCAO ischemia‐reperfusion. Combined with the results of our pilot research (*n* = 6 in each group, unpublished data), ononin (30 mg/kg) administered intraperitoneally 15 min before surgery was also safe and substantially mitigated unilateral nephrectomy‐induced hippocampal neuronal damage and cognitive deficits, as well as the accumulation of hippocampal pro‐inflammatory cytokines and MDA. Therefore, we finally chose the dosage of 30 mg/kg ononin for this study and injected it intraperitoneally into aged mice 15 min before surgery to further determine whether ononin could play a neuroprotective role against POCD.

It is essential to acknowledge some limitations in our study. First, we only explored the therapeutic potential of ononin in the early stage of POCD, therefore, further studies are needed to probe the role of ononin in long‐term cognitive dysfunction after surgery. Second, increasing evidence has demonstrated that anesthesia and/or surgery could cause neuroinflammation and cognitive decline in elderly mice (Qiao et al. 2023; Tao et al. 2014). Referring to many previous research designs (Terrando et al. 2010; Wang et al. 2023), we did not set up a “sham surgery” group as well because sham surgery is also a surgical trauma and requires anesthesia. In clinical practice, anesthesia and surgery are almost inseparable, so we mainly investigate the impacts of anesthesia and surgery in a group on postoperative cognitive function. Third, it is well known that there are many biological differences between humans and mice, so intraperitoneal injection of ononin (30 mg/kg) as a model in elderly mice cannot be extrapolated to humans. Fourth, although our results provide evidence that ononin may hinder or postpone the development of POCD, the precise molecular mechanisms remain poorly understood and need further investigation. Fifth, POCD models exhibit heterogeneity (e.g., varying degrees of surgical trauma, differences in anesthetic drugs), so further research is needed to validate the results' universality in other POCD models. Finally, to improve clinical relevance and translational value, future work will be devoted to determining the optimal dosage, administration methods, as well as the timing of ononin application in patients to diminish cognitive dysfunction induced by anesthesia/surgery.

## 5 Conclusion

In the present study, we demonstrated that pretreatment with ononin could rescue anesthesia/surgery‐induced neuronal damage and cognitive deficits in elderly mice, possibly through its anti‐inflammatory and antioxidant effects, which provides a valuable theoretical basis for its use in the prevention and treatment of postoperative cognitive impairment.

## Author Contributions

**Ming Li**: conceptualization, methodology, validation, writing – original draft, funding acquisition. **Qingmei Peng**: conceptualization, methodology, data curation, writing – original draft. **Min Zhu**: conceptualization, investigation, methodology, writing – original draft, project administration. **Qilin Liu**: writing – original draft, supervision, resources. **Simin Yang**: writing – original draft, resources. **Cansheng Gong**: resources, funding acquisition. **Jingyan Lin**: conceptualization, supervision, writing – original draft, writing – review and editing. **Qingbo Yu**: conceptualization, writing – original draft, writing – review and editing, supervision, software.

## Ethics Statement

All animal experiments were approved by North Sichuan Medical College Institutional Animal Care and Use Committee.

## Conflicts of Interest

The authors declare no conflicts of interest.

## Peer Review

The peer review history for this article is available at https://publons.com/publon/10.1002/brb3.70952

## Data Availability

All data are available from corresponding authors upon reasonable request.
